# Evaluation of Antibacterial Activity of Three Quaternary Ammonium Disinfectants on Different Germs Isolated from the Hospital Environment

**DOI:** 10.1155/2020/6509740

**Published:** 2020-12-11

**Authors:** Amal Ramzi, Bouchra Oumokhtar, Yassine Ez zoubi, Touria Filali Mouatassem, Moussa Benboubker, Abdelhakim El Ouali Lalami

**Affiliations:** ^1^Laboratory of Microbiology and Molecular Biology, Faculty of Medicine and Pharmacy Fez, University of Sidi Mohamed Ben Abdellah, Fez 30000, Morocco; ^2^Laboratory of Applied Organic Chemistry, Faculty of Sciences and Technology, Sidi Mohamed Ben Abdellah University, B.P. 2202-Route d'Imouzzer, Fez, Morocco; ^3^Biotechnology, Environmental Technology, and Valorization of Bio-resources Team, Department of Biology, Faculty of Science and Technology Al-Hoceima, Ajdir 32003, Abdelmalek Essaadi University, Tetouan, Morocco; ^4^Laboratory of Biotechnology and Preservation of Natural Resources, Sidi Mohamed Ben Abdellah University, Faculty of Sciences Dhar El Mahraz, Fez 30000, Morocco; ^5^Medical and Nursing Department, Hassan II University Teaching Hospital of Fez, Morocco; ^6^Higher Institute of Nursing Professions and Health Techniques of Fez, Regional Health Directorate, El Ghassani Hospital, Fez 30000, Morocco

## Abstract

**Background:**

The microbiological risk of the hospital environment, including inert surfaces, medical devices, and equipment, represents a real problem.

**Objective:**

This study is aimed at demonstrating and assessing the antibacterial activity of three synthetic disinfectants classified as quaternary ammoniums on different bacterial strains (Gram-negative and Gram-positive like *Escherichia coli*, *Klebsiella pneumoniae*, *Enterobacter cloacae*, *Pseudomonas aeruginosa*, *Acinetobacter baumannii*, and *Staphylococcus aureus*) isolated from the hospital environment. The reference strains included *Escherichia coli* ATCC 25922, *Staphylococcus aureus* ATCC 29213, and *Pseudomonas aeruginosa* ATCC 27853 used as negative control strains.

**Method:**

Three quaternary ammonium disinfectants were tested: DDN9® (0.5%) which contains didecylmethylpolyoxyethylammonium propionate as an active substance, spray (0.4%) containing quaternary ammonium compounds, and Phagosurf ND® (0.4%) with didecyldimethylammonium chloride. Their effect was evaluated using the disk diffusion technique and the broth dilution methods, allowing the Minimum Inhibitory Concentration (MIC) and then the Minimum Bactericidal Concentration (MBC).

**Result:**

Only the growth of Gram-positive bacteria and some strains of Gram-negative bacteria were inhibited by the three synthetic disinfectants. NDD9® demonstrated an antibacterial effect only against the Gram-positive strains (*S. aureus* and *S. aureus* ATCC 29213) with a MIC of 0.25 mg/ml. The disinfectant spray showed effect against all four strains including *E. coli* (9), *S. aureus*, *E. coli* ATCC 25922, and *P. aeruginosa* ATCC 27853 with an inhibitory concentration of 4 mg/ml, while the growth of *S. aureus* ATCC 29213 was inhibited at 2 mg/ml. The third disinfectant, Phagosurf ND®, inhibited only the growth of *S. aureus* ATCC 29213 at a MIC of 4 mg/ml.

**Conclusion:**

This study is the first here in Morocco to evaluate the bacterial activity of products intended for the control of the healthcare environment. The results obtained on the three disinfectants tested reveal an ineffectiveness against some isolated strains from the hospital environment.

## 1. Introduction

The patient's environment, particularly the hospital rooms and associated devices, is a vital source of multidrug-resistant pathogens that can be transmitted to other patients [1]. They constitute a place of interaction between patients with different pathologies, whether infectious or not, the caregivers, and the hospital environment [2]. Unless precautions are taken, they can lead directly into the healthcare environment, including contamination of air, water, devices, and surfaces [3].

However, surfaces and medical devices (e.g., stethoscopes, otoscopes, and thermometers) present a high contamination risk as they play a critical role in the cross-transmission of hospital microorganisms [2]. Otherwise, contamination may occur from medical equipment and objects that are not strictly considered medical devices. In this context, several studies have demonstrated the possible role of various contaminated items such as phones and computers in the transmission of infections [4, 5]. The prevalence of contamination is significantly related to the type of hospital units and the frequently used equipment. Indeed, El Ouali Lalami et al. [6] reported a predominance of bacterial strains in several departments with a prevalence of 19% in the Emergency Department. Oumokhtar et al. [7] have also assessed the contamination of the hospital environment in various sites in patients' rooms, including bed rails, bedside tables, toilets, door handles, room door handles, electrical knobs, cabinet knobs, and chemotherapy chair arms, by a variety of microorganisms. In the same contexts, other Moroccan studies have reported contamination of three departments, namely, the burns unit, the sterilization department, and the operating room, with varying rate prevalence of contamination (70%, 13%, and 7%, respectively). The same study showed that the autoclave, the bed rails, the bedside tables, and the operating tables are the most contaminated sites [8]. Some areas or equipment are more contaminated than others, while relevant research has shown a high prevalence of contamination of high contact surfaces and equipment around the patient's bed [9]. Meunier et al. [10] reported that medical devices and people close to patients were the most contaminated with pathogenic microorganisms.

In general, the presence of hospital microorganisms varies according to the units and medical devices used. Bacterial strains, including multiresistant species, play an important role in the hospital environment contamination. They can survive for a long time to physical, chemical, and environment, and they are isolated on different surfaces and in different equipment of the patient and care area [11]. They are the most common germs responsible for the infection, including healthcare-associated infections [12]. According to the literature, *S. aureus*, either MRSA or MRSA, *P. aeruginosa*, and *A. baumannii* are the bacteria most frequently isolated from inanimate surfaces and equipment in the patient area [13]. Also, numerous studies have reported a higher risk of contracting bacteria in long-stay rooms, which has occurred for both Gram-positive bacteria (*S. aureus*, *Enterococcus* species, and *Clostridium difficile*) and Gram-negative bacteria (*Acinetobacter* spp., *P. aeruginosa*, and *K. pneumoniae*) [14].

Various procedures have been adopted to prevent the risk of contamination by these pathogenic bacteria, including physical and chemical processes. However, disinfection with antimicrobial agents such as synthetic disinfectants (quaternary ammonium, halogenated compounds such as sodium hypochlorite, alcohols, peroxygen compounds such as hydrogen peroxide, and aldehydes such as glutaraldehyde) is still the most widely used method for disinfecting surfaces and materials in the healthcare environment [15]. Their use is significantly related to their selective toxicity against hospital microorganisms [16]. Consequently, the main objective of this study was to evaluate the antibacterial effect of quaternary ammonium disinfectants on different hospital environment-isolated strains. There have been few or no studies in the Fez region (north-central Morocco) that have studied the antibacterial effect of disinfectants on the hospital environment-isolated bacteria. The results of this study will provide an essential contribution to the healthcare-associated infection control committee. They will be of great interest to the hygiene services responsible for implementing preventive and corrective actions, particularly about the disinfection operations.

## 2. Material and Methods

### 2.1. Study Site

A prospective study was conducted at the Microbiology and Molecular Biology Laboratory of the Fez Faculty of Medicine and Pharmacy. This work consisted of testing the antibacterial activity of three synthetic disinfectants, from the quaternary ammonium class, frequently used at the University Hospital teaching Center (CHU) of Fez (north-east of Morocco, Lat: 34.°01°64°79, Ing: 4°98°44°95).

### 2.2. Bacterial Strains

The antibacterial activity of the tested disinfectants was evaluated on different bacterial strains such as Gram-negative *Escherichia coli* (*E. coli*) (9), *Klebsiella pneumoniae* (*K. pneumoniae*), and *Enterobacter cloacae* (*E. cloacae*), which are resistant to many beta-lactam antibiotics. In addition, other susceptible strains have also been tested such as *Escherichia coli* (E7) and *Acinetobacter baumannii* (*A. baumannii*). Also, a Gram-positive strain has been tested, namely, *Staphylococcus aureus* (*S. aureus*). These strains were collected on different surfaces in the hospital environment of the CHU of Fez (trolley, litter, incubator, cradle, mattress, table, etc.). The effect of disinfectants was evaluated on three reference strains, *Escherichia coli* ATCC 25922, *Staphylococcus aureus* ATCC 29213, and *Pseudomonas aeruginosa* ATCC 27853.

### 2.3. Disinfectants and Active Compounds Tested

Three disinfectants belonging to the family of quaternary ammoniums were selected to evaluate their antibacterial activity. These are the most used at the Fez University Hospital. Samples were collected from the central pharmacy of the center and met the requirements for correct storage conditions.

- Neutral disinfectant detergent (DDN9®): contains quaternary ammonium; didecylmethylpolyoxyethylammonium propionate as an active substance. It was concentrated at 0.5%
- Spray (0.4%): a surface disinfectant containing quaternary ammonium
- Phagosurf ND® at 0.4%: a disinfectant also belongs to the family of quaternary ammoniums based on didecyldimethylammonium chloride, whose molecule is a quaternary ammonium salt represented by a nitrogen atom linked to 4 alkyls, 2 methyl groups, and 2 decyl groups

### 2.4. Disk Diffusion Method

Antimicrobial activity was obtained using the disc diffusion method, according to CLSI [17]. Although it is recognized as reliable and reproducible, it is mainly used as a preliminary step in in-depth studies, as it gives access to essentially qualitative results. In short, a bacterial suspension previously adjusted with the 0.5 McFarland standard (approximately 10^8^ CFU/ml) was inoculated on Petri dishes containing Muller-Hinton Agar Medium (MHA). Sterilized discs (filter paper, 6 mm diameter) placed on the surface of each can were impregnated with 10 *μ*l of each disinfectant to be tested. Each test was performed in triplicate. After 24 hours of incubation at 37°C, the diameters of the inhibition zones were measured (mm).

### 2.5. The Broth Microdilution Method

This technique is highly recommended for water-soluble antimicrobial agents such as disinfectants. It acts as a reference method to define the target dilution of disinfectants for each species that corresponds to the Minimum Inhibitory Concentration (MIC).

#### 2.5.1. Determination of Minimum Inhibitory Concentration (MIC)

To determine the MIC, an inoculum was prepared, and a serial dilution of the three disinfectants was performed to give final concentrations ranging from 0.25 to 4 mg/ml. A volume of 200 *μ*l of each disinfectant dilution was added to each tube containing 1.8 ml of the bacterial suspension. All tubes were incubated at 37°C for 18-24 hours [18].

#### 2.5.2. Determination of the Minimum Bactericidal Concentration (MBC)

Referring to the MIC determination results, the MBC, which defined the bactericidal effect of disinfectant and expressed in *μ*g/ml or mg/ml, was determined by spreading from tubes without visible bacterial growth on MHA medium. After incubation at 37°C for 18 to 24 hours, the dishes showed no bacterial growth corresponding to the disinfectants' concentrations that represented the MBC [18].

### 2.6. Statistical Treatment

Statistical analysis of the antibacterial activity for all three disinfectants was performed using GraphPad Prism 8 software. A unidirectional analysis of variance (ANOVA) approach was used, and the results obtained are presented as means ± SD.

## 3. Results

### 3.1. Antibacterial Activity Testing of the Disinfectants against Bacterial Strains

The data in Table 1 showed that NMS9® disinfectant was effective against only two Gram-positive strains (*S. aureus* and *S. aureus* ATCC 29213), resulting in an inhibition zone diameter of 15 ± 0.33 mm and 13 ± 0.33 mm, respectively. On the other hand, Gram-negative bacteria have proven to be resistant to this disinfectant. It also showed that the spray was active against Gram-positive bacteria such as *S. aureus* and *S. aureus* ATCC 29213, giving an inhibition zone diameter of 13 ± 0.33 mm to 17 ± 0.33 mm, respectively. In added addition, this disinfectant only had efficacy against three Gram-negative bacteria, including *E. coli* (9), *E. coli* ATCC 25922, and *P. aeruginosa* ATCC 27853, while Phagosurf ND® is only active against Gram-positive *S. aureus* ATCC 29213 with an inhibitory diameter of 10 ± 0.57 mm.

### 3.2. Determination of Minimum Inhibitory Concentration (MIC) and the Minimum Bactericidal Concentration (MBC)

Results corresponding to the MIC (Table 2) showed that NDD9® only was effective on Gram-positive strains (*S. aureus* and *S. aureus* ATCC 29213). Indeed, it inhibited its growth at 0.25 mg/ml. While bacterial growth of the following four strains: *E. coli* (9), *S. aureus*, *E. coli* ATCC 25922, and *P. aeruginosa* ATCC 27853, had an inhibitory concentration of 4 mg/ml versus *S. aureus* ATCC 29213, their growth was inhibited at 2 mg/ml by the disinfectant spray. Furthermore, it was also noted that Phagosurf ND® only inhibited the growth of *S. aureus* ATCC 29213 at MIC 4 mg/ml.

Similar results were obtained for the minimum bactericidal concentration (MBC), which characterizes the bactericidal effect of the disinfectant (Table 2).

## 4. Discussion

Disinfection of environmental surfaces and medical devices in hospitals is essential. It remains the best process for preventing the transmission of microorganisms and, consequently, minimizing the risk of infection, especially with the significant emergence of antibiotic resistance. The risk is higher with the increasing incidence of antimicrobial resistance [19]. The results obtained in our study showed a variation in efficacy, which may be attributed to many factors; the action of the active molecule of each disinfectant, the genus, and the structure of the bacteria isolated developed the resistance or not to the antimicrobial agent and the adopted disinfection protocol. Depending on the spectrum of action, disinfectants could exert a biostatic effect by inhibiting bacterial growth or a bactericidal activity by killing and destroying microorganisms [20]. Nevertheless, some antimicrobial agents may potentially destroy only one class of microorganisms. This variation in the response to biocides depends on the physical-chemical characteristics of the microbial cell surface. When a disinfectant penetrates the cell wall, it can act on the pathogenic organism through mechanisms of coagulation and oxidation of microbial cell proteins, and it can act by denaturing bacterial enzymes [15]. The mechanism of action may interrupt the ability of bacteria to persist on environmental surfaces and develop resistance to certain chemicals or even antibiotics.

In general, many environmental variables could influence microbial viability and then affect the effectiveness of the disinfectant, for example, the presence of any organic matter (blood, serum, sputum, pus, and feces), which can indirectly play a role in enhancing the environmental resistance of microorganisms. This role is the interference of organic matter with the antimicrobial activity of disinfectants *via* chemical reactions resulting in a complex exhibiting less germicidal or nongermicidal properties and leaving a reduced quantity of active disinfectant agents [13]. Also, the specific characteristics of microorganisms such as genus, species, specific strain, ability to form a biofilm, and microorganism concentration constitute the principal factors in a nosocomial pathogen's capacity to survive on inanimate surfaces and equipment. The environmental factors, including UV radiation, temperature, humidity, organic material, and surface type, may also affect the survival of pathogens [21–23]. Indeed, the biological factor of making biofilm increases recently, which leads to the rise of resistance to disinfectants. That is supposed to be due to the many factors: production of exopolymeric substances (EPS) surrounding the bacteria. The EPS reduces the penetration of biocides into the biofilm, inactivates some disinfectants by binding to them, and inactivates some disinfectants by liberating enzymes. The biofilm can be composed of various microorganisms' species forming a polymicrobial biofilm with higher resistance to disinfectants than monospecies biofilms [24]. This increased resistance mechanism may result from an increased disinfectant inactivation due to a more complex EPS or from the shielding of sensitive organisms by externally situated disinfectant tolerant organisms [25].

Disinfectants based on quaternary ammonium constitute an excellent antimicrobial agent due to their significant biocide activity, long-term durability, and compatibility with the environment [26]. However, the inappropriate operation could lead to the adaptation of bacteria to these disinfectants and increase resistant strains' emergence by developing resistance genes (qac gene: quaternary ammonium compounds). That causes preeminent problems [27]. These compounds are generally more effective on Gram-positive bacteria than on Gram-negative bacteria. Generally, Ioannou et al. [28] have demonstrated the efficacy of disinfectant quaternary ammonium compounds against *Staphylococcus aureus*. They have physicochemical properties by having two poles, a hydrophobic pole and another positively charged hydrophilic pole, allowing the molecule to adsorb to inert surfaces, which gives this type of molecule detersive properties in addition to its bactericidal activity. These molecules can also adsorb irreversibly to phospholipids and proteins in the bacterial membrane; this adsorption leads to changes in permeability and then damages the cytoplasmic membrane leading to leakage of constituents (particularly potassium ions) [29].

Regarding the result that some disinfectants are effective on some antibiotic-resistant strains, the spray in our study against resistant bacteria (*E.coli* (9) resistant to beta-lactam) could be attributed to the high concentration used (4 mg/ml). The disinfectant action inside the bacteria and perhaps this strain developed just resistance to antibiotics and not yet to the disinfectant used. It has been demonstrated that methicillin-resistant *Staphylococcus aureus* and vancomycin-resistant *Enterococcus* were reduced by disinfection operation to the rooms previously occupied by patients colonized by the same pathogen [30, 31].

Thus, the efficacy of disinfectants is positively linked to various factors. Nevertheless, the protocol adopted for applying disinfectant and the correct use are essential because even using a suitable disinfectant can be ineffective if the application method is not proper (the use of the disinfectant at the incorrect dilution, the nonrespect of disinfectant contact time, the regular use of the same disinfectant at the same dose, which could lead to the appearance of resistance, and also the use of some disinfectants on inappropriate surfaces, for example, surfaces where biofilms could develop and could reduce the effectiveness of the disinfectants) [15]. The use of disinfectants frequently or improperly could also create a serious problem [32]. Moreover, it is crucial to consider that the biocide activity is influenced by the concentration, time of contact, and potential traces of interfering material or substance: organic fluids, soap, metallic ions, and pH [33–35]. Choosing the right product is also crucial since a low disinfectant will not be effective even if appropriately applied [15]. Besides, quaternary ammoniums remain effective even in organic matter or in hard water [36]. To be highly effective, they can be combined with nonionic agents. For obtaining a disinfectant having a broad spectrum, it is recommended to make a minimum combination of three different types of quaternary ammoniums [25].

Recently, the surfaces of some hospital services in the city of Fez were found contaminated by many microorganisms such as coagulase-negative *Staphylococcus*, *Bacillus sp.*, *Staphylococcus aureus*, *Aeromonas salmonicida*, *Escherichia coli*, *Pseudomonas aeruginosa*, *Pseudomonas vesicularis*, *Acinetobacter baumannii*, and *Klebsiella sp.* [3, 6, 7, 37]. Therefore, it would be very interesting to evaluate other disinfectants' effectiveness and explore other alternatives based on natural products.

## 5. Conclusion

The present study highlighted the effect of some disinfectants based on the quaternary ammonium in several bacterial strains (antibiotic-sensitive and antibiotic-resistant). The results showed the variable effectiveness of the disinfectants tested. Indeed, NDD9® has an antibacterial effect only against Gram-positive strains. However, the disinfectant spray was effective against certain positive and negative strains. In contrast, Phagosurf ND® inhibited the development of only one positive strain. Therefore, it is necessary to reconsider the factors that affect the effectiveness of these synthetic products in the hospital disinfection process. The use of the same products with the same active substances and perhaps at the same doses for a long period makes the germs increasingly resistant. It is therefore recommended to regularly use other disinfectants in different concentrations. Nevertheless, further studies are required to evaluate other disinfectants' antibacterial potential with other bioactive molecules or evaluate the combinatory effect of some disinfectants to improve their antimicrobial efficacy. The results of this study will provide an essential contribution to the healthcare-associated infection control committee, and they will be of great interest to the hygiene departments responsible for carrying out preventive and corrective actions, in particular as regards the environment and surface risk management.

## Figures and Tables

**Table 1:** Antibacterial activity of the three disinfectants on the tested strains.

The strains tested	Inhibition zone of disinfectant (mm)		
	DDN9®	Spray	Phagosurf ND®
*E. coli* (7)	R	R	R
*K. pneumoniae*	R	R	R
*E. coli* (9)	R	9 ± 0.33	R
*E. cloacae*	R	R	R
*P. aeruginosa*	R	8 ± 0.33	R
*A. baumannii*	R	8 ± 0.33	R
*S. aureus*	15 ± 0.33	13 ± 0.33	8 ± 0.33
*E. coli* ATCC 25922	R	9 ± 0.00	R
*S. aureus* ATCC 29213	13 ± 0.33	17 ± 0.33	10 ± 0.57
*P. aeruginosa* ATCC 27853	R	11 ± 0.33	R

R: resistance; data are the mean of three replicates and are represented as mean ± standard deviation.

**Table 2:** Minimum Inhibitory Concentration (MIC) and Minimum Bactericidal Concentration (MBC) of the three disinfectants tested against many resistant and susceptible strains.

	MIC (mg/ml)			MBC (mg/ml)		
	DDN9®	Spray	Phagosurf ND®	DDN9®	Spray	Phagosurf ND®
*E. coli* (7)	—	—	—	—	—	—
*K. pneumoniae*	—	—	—	—	—	—
*E. coli* (9)	—	4	—	—	4	—
*E. cloacae*	—	—	—	—	—	—
*P. aeruginosa*	—	—	—	—	—	—
*A. baumannii*	—	—	—	—	—	—
*S. aureus*	0.25	4	—	0.25	4	—
*E. coli* ATCC25922	—	4	—	—	4	—
*S. aureus* ATCC29213	0.25	2	4	0.25	2	4
*P. aureus* ATCC27853	—	4	—	—	4	—

## Data Availability

The data used in this study are included in the article.
